# Supplementary material for: Meta-Analysis and Gene Set Enrichment Relative to ER Status Reveal Elevated Activity of MYC and E2F in the “Basal” Breast Cancer Subgroup
Source: PLoS One. 2009 Mar 9;4(3):e4710. doi: 10.1371/journal.pone.0004710 (PMC2650420; doi:10.1371/journal.pone.0004710)
Supplement: Dataset S1 — GSEA results from first screen. The file contains a zipped archive of the HTML results of the second GSEA screen. Reports for enriched or depleted datasets may be accessed from the “index.html” link within the my_analysis.GseaPreranked.1219975950625.rpt.zip ZIP archive. Enrichment plots are available for each of the 50 most enriched and depleted gene sets. (13.32 MB ZIP) [file pone.0004710.s001.zip › my_analysis.GseaPreranked.1219975950625.rpt.zip!//CELL_CYCLE.html]

Details for gene set CELL\_CYCLE[GSEA]

|  || Dataset | zSco.ERneg.to.ERpos\_collapsed |
| Phenotype | NoPhenotypeAvailable |
| Upregulated in class | na\_pos |
| GeneSet | CELL\_CYCLE |
| Enrichment Score (ES) | 0.64425045 |
| Normalized Enrichment Score (NES) | 2.5225356 |
| Nominal p-value | 0.0 |
| FDR q-value | 0.0 |
| FWER p-Value | 0.0 |
Table: GSEA Results Summary

  

Fig 1: Enrichment plot: CELL\_CYCLE      
 Profile of the Running ES Score & Positions of GeneSet Members on the Rank Ordered List

  

| PROBE | GENE SYMBOL | GENE\_TITLE | RANK IN GENE LIST | RANK METRIC SCORE | RUNNING ES | CORE ENRICHMENT || 1 | E2F3 |  |  | 6 | 7.369 | 0.0315 | Yes |
| 2 | CDC20 |  |  | 9 | 7.151 | 0.0624 | Yes |
| 3 | CCNE1 |  |  | 24 | 6.716 | 0.0905 | Yes |
| 4 | MCM5 |  |  | 31 | 6.533 | 0.1184 | Yes |
| 5 | HDAC2 |  |  | 32 | 6.511 | 0.1466 | Yes |
| 6 | ORC6L |  |  | 33 | 6.507 | 0.1749 | Yes |
| 7 | BUB1 |  |  | 41 | 6.445 | 0.2023 | Yes |
| 8 | CCNA2 |  |  | 52 | 6.297 | 0.2289 | Yes |
| 9 | CDC45L |  |  | 108 | 5.824 | 0.2500 | Yes |
| 10 | CCNA1 |  |  | 117 | 5.732 | 0.2743 | Yes |
| 11 | SKP2 |  |  | 127 | 5.694 | 0.2983 | Yes |
| 12 | MCM4 |  |  | 180 | 5.436 | 0.3180 | Yes |
| 13 | MCM6 |  |  | 198 | 5.317 | 0.3398 | Yes |
| 14 | ORC1L |  |  | 212 | 5.280 | 0.3617 | Yes |
| 15 | PTTG1 |  |  | 225 | 5.206 | 0.3834 | Yes |
| 16 | CHEK1 |  |  | 253 | 5.088 | 0.4034 | Yes |
| 17 | BUB1B |  |  | 271 | 5.016 | 0.4239 | Yes |
| 18 | CDC25A |  |  | 281 | 4.993 | 0.4449 | Yes |
| 19 | MCM7 |  |  | 289 | 4.970 | 0.4659 | Yes |
| 20 | CDKN2A |  |  | 330 | 4.858 | 0.4840 | Yes |
| 21 | CDC25B |  |  | 340 | 4.826 | 0.5043 | Yes |
| 22 | CCNB2 |  |  | 431 | 4.589 | 0.5174 | Yes |
| 23 | MAD2L1 |  |  | 477 | 4.446 | 0.5333 | Yes |
| 24 | MCM2 |  |  | 630 | 4.080 | 0.5395 | Yes |
| 25 | MCM3 |  |  | 656 | 4.019 | 0.5551 | Yes |
| 26 | TFDP1 |  |  | 750 | 3.843 | 0.5647 | Yes |
| 27 | PTTG3 |  |  | 766 | 3.813 | 0.5801 | Yes |
| 28 | CDC7 |  |  | 769 | 3.807 | 0.5965 | Yes |
| 29 | CHEK2 |  |  | 791 | 3.790 | 0.6114 | Yes |
| 30 | PRKDC |  |  | 1234 | 3.204 | 0.5919 | Yes |
| 31 | CDC2 |  |  | 1259 | 3.182 | 0.6039 | Yes |
| 32 | PCNA |  |  | 1346 | 3.087 | 0.6108 | Yes |
| 33 | CCNB1 |  |  | 1626 | 2.823 | 0.6020 | Yes |
| 34 | E2F1 |  |  | 1695 | 2.764 | 0.6089 | Yes |
| 35 | PKMYT1 |  |  | 1715 | 2.753 | 0.6194 | Yes |
| 36 | CDC25C |  |  | 1794 | 2.700 | 0.6252 | Yes |
| 37 | CDK6 |  |  | 1932 | 2.584 | 0.6261 | Yes |
| 38 | E2F4 |  |  | 1948 | 2.572 | 0.6361 | Yes |
| 39 | ATM |  |  | 1994 | 2.536 | 0.6437 | Yes |
| 40 | HDAC6 |  |  | 2163 | 2.411 | 0.6415 | Yes |
| 41 | CCND2 |  |  | 2506 | 2.197 | 0.6252 | Yes |
| 42 | CDK4 |  |  | 2546 | 2.176 | 0.6317 | Yes |
| 43 | ORC3L |  |  | 2573 | 2.160 | 0.6391 | Yes |
| 44 | CDK2 |  |  | 2688 | 2.099 | 0.6396 | Yes |
| 45 | CCND3 |  |  | 2854 | 2.011 | 0.6359 | Yes |
| 46 | HDAC4 |  |  | 2922 | 1.973 | 0.6394 | Yes |
| 47 | WEE1 |  |  | 3048 | 1.909 | 0.6382 | Yes |
| 48 | E2F5 |  |  | 3105 | 1.885 | 0.6422 | Yes |
| 49 | CDC6 |  |  | 3185 | 1.847 | 0.6443 | Yes |
| 50 | ORC5L |  |  | 3377 | 1.760 | 0.6375 | No |
| 51 | CCNE2 |  |  | 3424 | 1.738 | 0.6415 | No |
| 52 | ORC4L |  |  | 4538 | 1.307 | 0.5632 | No |
| 53 | PTTG2 |  |  | 4656 | 1.267 | 0.5599 | No |
| 54 | HDAC1 |  |  | 4997 | 1.157 | 0.5392 | No |
| 55 | E2F2 |  |  | 5382 | 1.019 | 0.5146 | No |
| 56 | TGFB1 |  |  | 5452 | 0.993 | 0.5137 | No |
| 57 | MDM2 |  |  | 5816 | 0.872 | 0.4901 | No |
| 58 | ESPL1 |  |  | 5874 | 0.853 | 0.4895 | No |
| 59 | CDC14A |  |  | 6609 | 0.618 | 0.4368 | No |
| 60 | ORC2L |  |  | 6907 | 0.517 | 0.4166 | No |
| 61 | TP53 |  |  | 7848 | 0.167 | 0.3464 | No |
| 62 | ABL1 |  |  | 7913 | 0.141 | 0.3422 | No |
| 63 | RB1 |  |  | 8000 | 0.105 | 0.3361 | No |
| 64 | E2F6 |  |  | 8656 | -0.155 | 0.2874 | No |
| 65 | GADD45A |  |  | 8982 | -0.305 | 0.2641 | No |
| 66 | GSK3B |  |  | 9187 | -0.398 | 0.2505 | No |
| 67 | HDAC5 |  |  | 9206 | -0.408 | 0.2509 | No |
| 68 | MAD1L1 |  |  | 9406 | -0.516 | 0.2381 | No |
| 69 | BUB3 |  |  | 9557 | -0.594 | 0.2294 | No |
| 70 | CDKN1A |  |  | 10787 | -1.380 | 0.1426 | No |
| 71 | HDAC3 |  |  | 10972 | -1.548 | 0.1354 | No |
| 72 | HDAC7A |  |  | 11335 | -1.861 | 0.1161 | No |
| 73 | CDKN1B |  |  | 12472 | -3.218 | 0.0444 | No |
| 74 | CCNH |  |  | 13015 | -4.513 | 0.0230 | No |
Table: GSEA details [plain text format]

  

Fig 2: CELL\_CYCLE: Random ES distribution      
 Gene set null distribution of ES for **CELL\_CYCLE**
